# Multi-isotope analysis reconstructs termite feeding in chimpanzees

**DOI:** 10.1038/s41598-026-45049-4

**Published:** 2026-04-28

**Authors:** Sven Brömme, Vicky M. Oelze, Alfredo Martínez-García, Jennifer N. Leichliter, Gerald H. Haug, Hubert B. Vonhof, Fiona A. Stewart, Alex K. Piel, Tina Lüdecke

**Affiliations:** 1 Emmy Noether Group for Hominin Meat Consumption, Max Planck Institute for Chemistry, Mainz, Germany; 2 Department of Anthropology, University of Santa Cruz, Santa Cruz, CA USA; 3 Department of Climate Geochemistry, Max Planck Institute for Chemistry, Mainz, Germany; 4 Department of Earth Sciences, ETH Zürich, Zürich, Switzerland; 5 Department of Human Origins, Max Planck Institute for Evolutionary Anthropology, Leipzig, Germany; 6 Department of Anthropology, University College London, London, UK

**Keywords:** Isotope ecology, Trophic level reconstruction, Food webs, Human evolution, Primates, Ecology, Ecology, Evolution, Zoology

## Abstract

Multi-isotope analyses of extant and extinct animals provide key insights into dietary ecology. Here, we examined mineral-bound nitrogen isotopes alongside carbonate carbon and oxygen stable isotope ratios in tooth enamel from sympatric chimpanzees, other primates (yellow baboons, red-tailed, and red colobus monkeys), herbivores, omnivores, and a radicivore from the miombo woodlands of the Issa Valley, Tanzania. Chimpanzees occupy a distinct isotopic niche characterized by low nitrogen and carbon isotope values and enriched oxygen isotope values relative to other primates. This unique isotopic niche likely reflects their reliance on termites as a major source of dietary protein. Our data indicate that termites contribute substantially to the chimpanzee’s nitrogen intake (at least 50%), consistent with nitrogen isotope values of chimpanzees. These findings from woodland-dwelling primates demonstrate the power of multi-isotope approaches for reconstructing modern and past dietary ecology and highlight their potential for interpreting ancient diets, including those of hominins.

**Supplementary Information:**

The online version contains supplementary material available at 10.1038/s41598-026-45049-4.

## Introduction

Stable isotope analysis provides an integrated record of dietary behavior over months to years and is a powerful tool for reconstructing feeding ecology, physiology, and environmental context in both modern and fossil animals. Because the behavior of extinct species cannot be directly observed, modern ecosystems offer essential reference frameworks for interpreting fossil isotope data and tracing evolutionary adaptations in diet and behavior^1,2^. Such analogues are particularly valuable for understanding the ecology of hominins, as some modern primates share deep evolutionary histories and key physiological and ecological traits with early hominins. Chimpanzees (*Pan troglodytes*) – and to a lesser extent some baboon taxa (*Papio* spp.) – approximate hominin body size (e.g., *Sahelanthropus*, *Ardipithecus*, *Australopithecus*)^3,4^, and chimpanzees exhibit complex foraging behaviors also attributed to hominins, including the ability to select and modify raw materials to produce tools for specific tasks such as termite fishing or nut cracking^5^. These behaviors provide insight into the cognitive and ecological foundations of tool use and diet in our lineage^6–8^.

Isotopic studies of modern ecosystems typically analyze biological materials, including body fluids, soft tissues, or collagen from bone or dentin^9,10^. However, these materials are rarely preserved over geologic timescales, making diagenetically robust tooth enamel a preferred archive for paleodietary reconstructions. While stable carbon (δ¹³C_enamel_) and oxygen (δ¹⁸O_enamel_) isotope values have been routinely measured in the inorganic fraction of enamel for decades, nitrogen isotope (δ¹⁵N_enamel_) analysis has been far more challenging because enamel contains only trace amounts of nitrogen. This small nitrogen fraction has historically been difficult to extract and measure, limiting δ^15^N_enamel_ applications in fossil teeth despite enamel’s excellent preservation potential^11,12^. Recent methodological advances now allow δ¹⁵N_enamel_ to be measured reliably from enamel’s organic fraction^13^, greatly expanding the potential to link isotopic patterns in modern ecosystems to reconstructions of trophic ecology of the past^14–18^.

Nitrogen isotope ratios of body tissues are enriched relative to diet due to the excretion of ^15^N-depleted nitrogenous waste products, primarily ammonium, urea and uric acid, as well as feces and sweat^2^. This enrichment – typically 3 to 6‰ – has been quantified in experimental as well as ecological studies^13,17–20^ and permits reconstruction of trophic position from tissues and enamel.

Complementary, stable carbon isotopes in animal tissues are a well-established tool for reconstructing plant based-diets^21^. In African savanna ecosystems, which often contain a mosaic of plant types, δ^13^C values are primarily influenced by the proportion of C_3_ versus C_4_ plants consumed. Most dicots (such as trees and shrubs) use the C_3_ photosynthetic pathway, which results in δ^13^C values ranging from −22 to −37‰, with an average of −27‰^22^. Monocots (primarily grasses), which are well adapted to warm, arid environments, use the C_4_ photosynthetic pathway and typically have δ^13^C values ranging from −9 to −15‰, with an average of −12.5‰. A fractionation of ca. +14‰ between diet and enamel has been observed for large herbivores^23,24^ with a slightly smaller fractionation of +11.8‰ documented for chimpanzees^25^.

Oxygen isotopes primarily reflect the δ¹⁸O of body water, which is influenced by the isotopic composition of drinking, food, and metabolic water. These values vary with environmental factors such as temperature, rainfall, altitude, and evaporation, as well as species-specific drinking behavior. In seasonal environments, δ¹⁸O_enamel_ profiles can also record wet-dry cycles during tooth formation^26–28^.

**Fig. 1:**
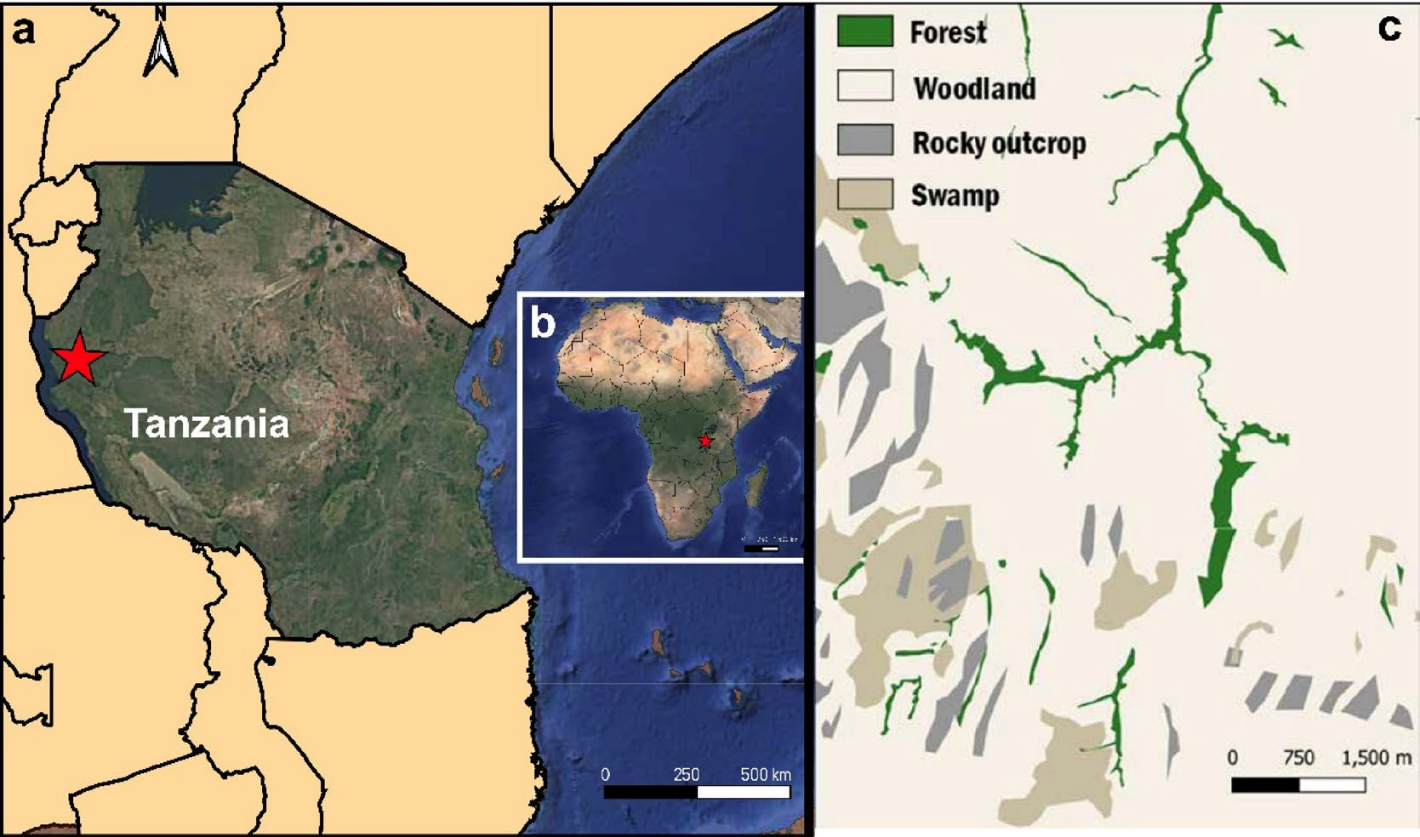
Location of Issa Valley (red star) in western Tanzania (**a**), location on the African continent (**b**), and vegetation zones^31^(**c**).

By understanding how δ^15^N_enamel_, δ^13^C_enamel_, and δ^18^O_enamel_ values vary with diet, trophic position, water sources, and physiological processes, we can interpret the isotopic niches of different taxa within their ecological and environmental contexts, using modern primates as analogs for reconstructing the dietary behavior of extinct hominids^29,30^. To explore these relationships, we focused on the Issa Valley in western Tanzania, a savanna-woodland mosaic of open C_4_-dominated habitats interspersed with C_3_-rich riparian forest^31^(Fig. 1). Paleohabitat reconstructions suggest that such habitats resemble those inhabited by hominids during key periods in early human evolution^32–34^, making Issa an ideal modern analog for investigating diet-environment interactions. Reconstructing hominid paleodiets requires integrated isotopic data for consumers and their environment. Here, we analyze nitrogen, carbon, and oxygen isotopes derived from tooth enamel of sympatric wild eastern chimpanzees (Pan troglodytes schweinfurthii), yellow baboons (Papio cynocephalus), and other mammalian fauna from Issa to evaluate how enamel isotopes reflect dietary and ecological patterns across taxa. Chimpanzees in this community have been the focus of long-term research addressing key questions about their feeding ecology. Previous studies combined stable isotope analyses of chimpanzee soft tissues with δ^15^N data from locally collected plants^35^ to investigate trophic enrichment compared to their diet.

Primates play an important ecological role in ecosystems, often influencing plant dispersal and habitat structure. The eastern chimpanzee diet is highly seasonal: during the wet season, Issa chimpanzees rely heavily on ripe fruits, seeds, flowers and leaves from at least 69 identified plant species, primarily sourced from within the riparian forests; whereas in the dry season, they turn to unripe seed pods from miombo woodlands^35^. At Issa, as elsewhere, chimpanzees are largely frugivorous, but they also use flexible probe tools to fish for termites, particularly for *Macrotermes subhyalinus*^36^. Individual chimpanzees may engage in termite fishing for extended periods of time during the rainy season, when soldier termites are particularly active near the mound surface^37–39^. Termite consumption and termite fishing is a low-risk protein resource that is regularly consumed, compared to vertebrate meat, which is also protein rich, but eaten more rarely.

Issa chimpanzees also consume mammalian prey, both from opportunistic scavenging and active hunting^40,41^. Prior to habituation of the Issa community in 2018, evidence of meat consumption based on fecal analysis was limited, despite chimpanzees routinely encountering potential prey^42^. Since habituation, hunts of small- to mid-sized vertebrate prey have been observed, as well as a single case of carcass theft from a crowned eagle^40^. These behaviors suggest that, while meat is not a regular dietary staple at Issa, it is selectively and opportunistically incorporated into their diet. While these observations provide useful information about the Issa chimpanzee diet, isotopic data can complement and quantify dietary components in a multiyear record, even in retrospect.

Here we integrate multi-isotope data from enamel and ecological observations, demonstrating the utility of δ^15^N_enamel_ in combination with δ^13^C_enamel_ and δ^18^O_enamel_ for reconstructing and quantifying dietary behavior in extant primates. Our study aims to not only demonstrate that stable isotope analysis of primate tooth enamel offers a reliable proxy for reconstructing feeding behavior and trophic ecology in modern primates, but also quantify different dietary components, which are essential for the nitrogen budget. Our findings offer an important comparative dataset which can aid in interpretation of isotopic niches derived from the fossil tooth enamel of hominids and hominins.

## Results

Inorganic δ^13^C_enamel_ and δ^18^O_enamel_ values of the carbonate (CO_3_) phase of enamel, and δ^15^N_enamel_ values of enamel-bound organic matter were determined for fauna with diverse dietary niches (i.e. grazers, browsers, omnivores, radicivores, primates) alongside sympatric yellow baboons and eastern chimpanzees from the Issa Valley, Tanzania (Table 1). We present δ^13^C_enamel_, δ^15^N_enamel_, and δ^18^O_enamel_ data for all analyzed specimens (*n* = 45), followed by biplots of δ^13^C_enamel_/δ^15^N_enamel_, δ^18^O_enamel_/δ^15^N_enamel_, and δ^13^C_enamel_/δ^18^O_enamel_.

### Nitrogen isotope values

**Fig. 2:**
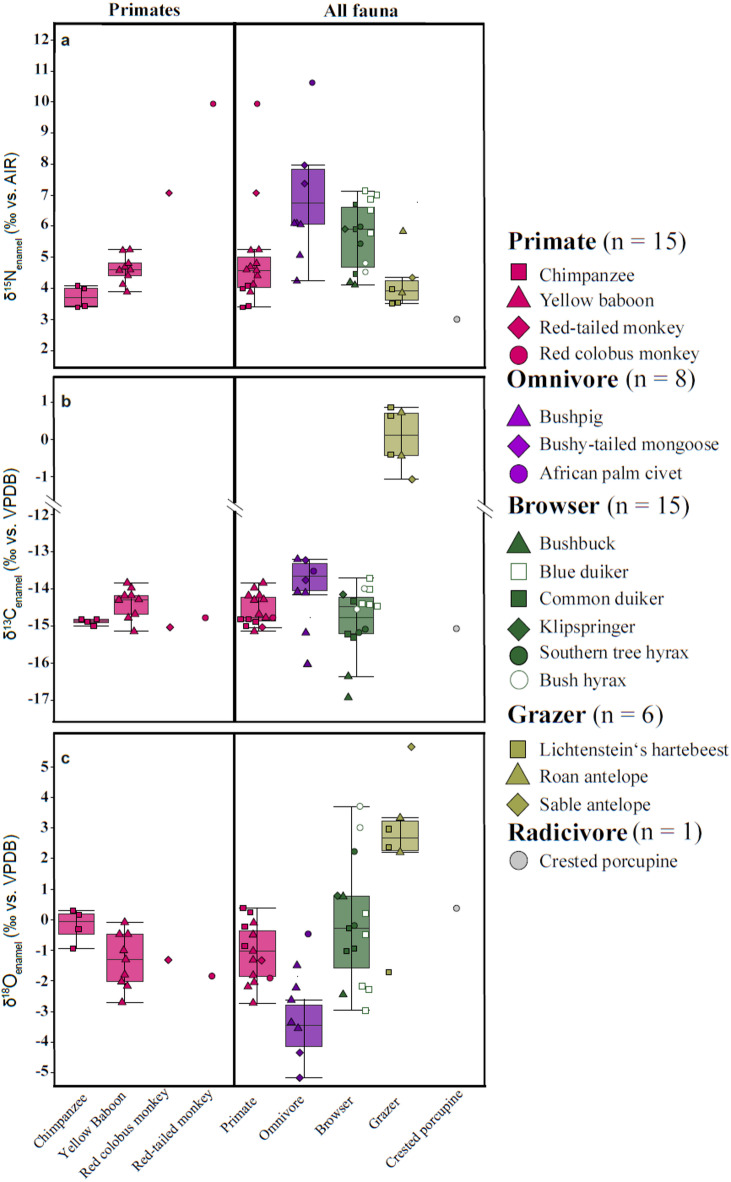
Isotopic values of the Issa Valley fauna: (**a**) δ^15^N_enamel_, (**b**) δ^13^C_enamel_, (**c**) δ^18^O_enamel_. Boxplots on the right show values grouped by ecological category, while those on the left display only the primate data, separated by taxon for better visualization. Boxplots show interquartile ranges with medians indicated as solid lines. Among primates, chimpanzees exhibit the lowest δ^15^N_enamel_ values, overlapping with grazing herbivores. Baboons have the highest median δ^13^C_enamel_, consistent with regular C_4_ resource utilization in open woodlands. The largest variation in δ^18^O_enamel_ values occurs among forest-dwelling browsers. Note there is a break in the in δ^13^C_enamel_ axis between − 12 and − 1‰.

**Fig. 3:**
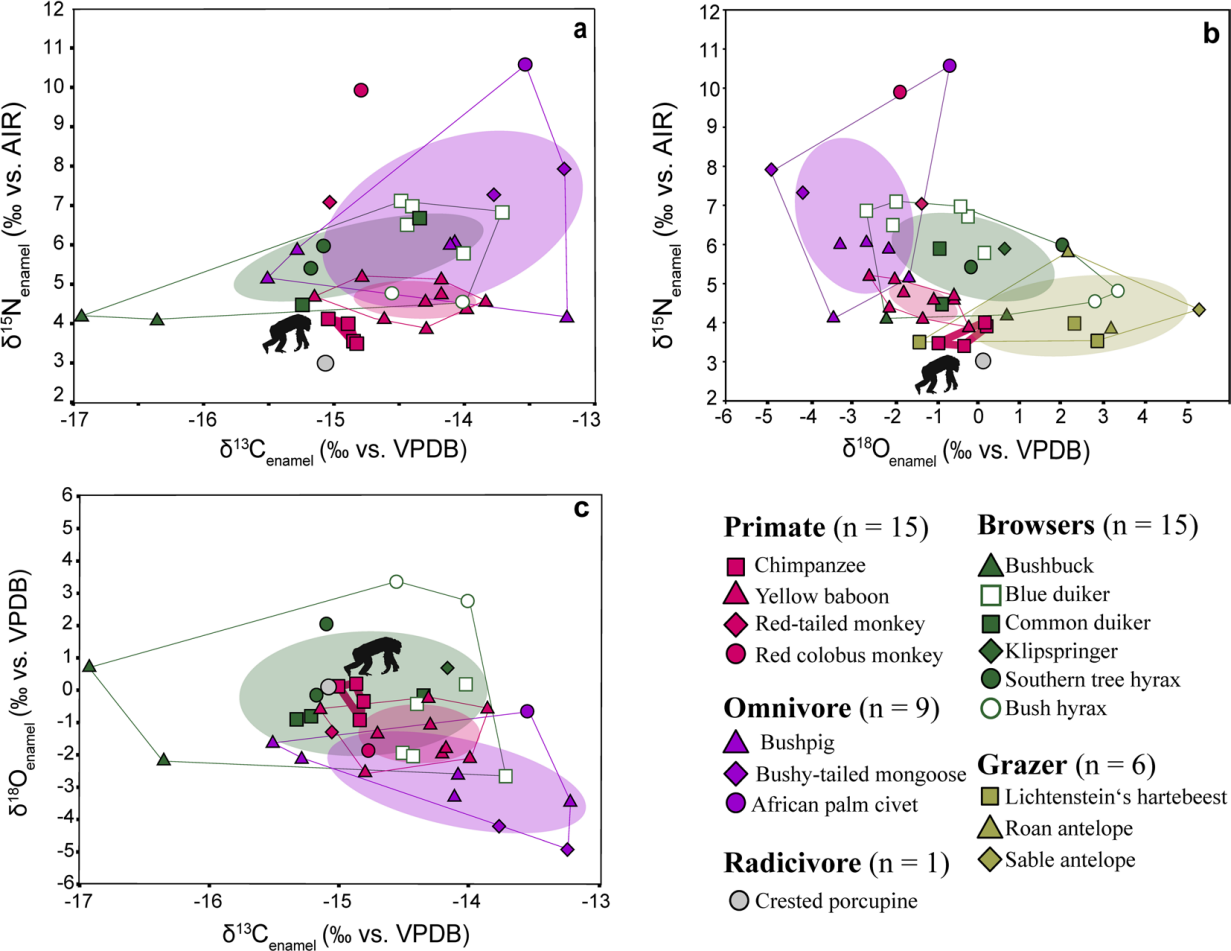
Biplots of (**a**) δ^15^N_enamel_ vs. δ^13^C_enamel_, (**b**) δ^15^N_enamel_ vs. δ^18^O_enamel_, and (**c**) δ^18^O_enamel_ vs. δ^13^C_enamel_ of Issa Valley fauna. Ellipses indicate 40% SEA_C_, convex hulls encompass full variation for each group (lines). Due to limited sample size (*n* < 5) for most primate taxa, SEA_C_ was calculated only for baboons. Chimpanzees’ isotopic niche is presented as a convex hull only. Individual data points for red-tailed and red colobus monkeys are displayed but excluded from statistical analyses. Note that grazers are not shown in panels (**a**) and (**c**) due to distinct δ^13^C_enamel_ values; but extended plots including this group are provided in Supplementary Figure S2.

The δ^15^N_enamel_ values of all specimens ranged from 3.0 to 10.6‰ (Fig. 3a), with an overall median ($\tilde{\mathrm{x}}$) of 4.8 ± 1.6‰ (H(3) = 21.25, *p* < 0.001). Median values were 4.6 ± 1.6‰ for primates, 5.9 ± 1.0‰ for browsers, 6.1 ± 2.0‰ for omnivores, 3.9 ± 0.9‰ for grazers, and 3.0‰ for the single radicivore (Fig. 2a). Primates had statistically significant lower δ^15^N_enamel_ values compared to omnivores (*p* = 0.001) and browsers (*p* = 0.005), but similar values to grazers (*p* = 0.258). The δ^15^N_enamel_ values among omnivores and browsers were similar (*p* = 0.359), whereas grazers showed lower ones (*p* ≤ 0.001).  

Primate data were further subdivided by genus to visualize potential isotopic similarities or differences between baboons and chimpanzees (see Fig. 3): δ^15^N_enamel_ values of chimpanzees ($\tilde{\mathrm{x}}$  = 3.7 ± 0.4‰) were significantly lower compared to baboons ($\tilde{\mathrm{x}}$ = 4.6 ± 0.4‰, *p* = 0.002), as well as the single analyzed red colobus (9.9‰) and red-tailed monkey (7.5‰).

**Table 1:** List of all analyzed specimens including group, common and scientific name, catalog ID, and enamel nitrogen, carbon and oxygen values in permille (‰). Mean values for each group are calculated below each section. Nitrogen content is reported in the Supplementary Material.

Group		Common name		Scientific name	ID (ISA)	δ^15^*N*_enamel_(‰ vs. AIR)	δ^13^C_enamel_(‰ vs. VPDB)	δ^18^O_enamel_(‰ vs. VPDB)
Primate		Yellow baboon		*Papio cynocephalus*	33	4.4	−14.0	−2.1
					34	3.9	−14.3	−0.2
					35	4.7	−15.2	−0.6
					36	4.6	−13.8	−0.6
					37	5.2	−14.8	−2.6
					38	4.6	−14.3	−1.0
					39	4.8	−14.2	−1.8
					40	4.1	−14.7	−1.3
					41	5.1	−14.2	−2.0
		Eastern chimpanzee		*Pan troglodytes schweinfurthii*	42	3.4	−14.8	−0.3
					43	4.1	−15.0	0.1
					44	4.0	−14.9	0.2
					46	3.4	−14.8	−0.9
		Red colobus monkey		*Piliocolobus tephrosceles*	24	9.9	−14.8	−1.9
		Red-tailed monkey		*Cercopithecus ascanius*	26	7.1	−15.0	−1.3
**Primate mean values (n = 15)**						**4.6 ± 1.6**	**−14.8 ± 0.4**	**−1.0 ± 0.9**
Omnivore (generalist)		Bushpig		*Potamochoerus larvatus*	17	6.1	−14.1	−2.6
					18	4.2	−13.2	−3.4
					19	5.9	−15.3	−2.1
					20	5.2	−15.5	−1.6
					21	6.0	−14.1	−3.3
Omnivore (insectivore)		Bushy-tailed mongoose		*Bdeogale crassicauda*	22	7.4	−13.8	−4.2
					23	7.9	−13.2	−4.9
Omnivore (frugivore)		African palm civet		*Nandinia binotata*	25	10.6	−13.5	−0.7
**Omnivore mean values (n = 8)**						**6.1 ± 2.0**	**−13.9 ± 0.9**	**−2.9 ± 1.4**
Radicivore		Crested porcupine		*Hystrix cristata*	12	3.0	−15.1	0.1
**Radicivore mean values (n = 1)**						**3.0**	**−15.1**	**0.1**
Browser		Bushbuck		*Tragelaphus scriptus*	08	4.1	−16.4	−2.2
					09	4.2	−16.9	0.7
		Common duiker		*Sylvicapra grimmia*	10	5.9	−15.3	−0.9
					11	4.5	−15.2	−0.8
					27	6.7	−14.3	−0.2
		Blue duiker		*Philantomba monticola*	28	5.8	−14.0	0.2
					29	6.9	−13.7	−2.7
					30	7.1	−14.5	−1.9
					31	7.0	−14.4	−0.4
					32	6.5	−14.4	−2.0
		Southern tree hyrax		*Dendrohyrex arboreus*	13	5.4	−15.2	−0.2
					14	6.00	−15.1	2.0
		Bush hyrax		*Heterohyrax brucei*	15	4.8	−14.6	3.4
					16	4.5	−15.00	2.7
		Klipspringer		*Oreotragus oreotragus*	07	5.9	−14.2	0.7
**Browser mean values (n = 15)**						**5.9 ± 1.0**	**−14.5 ± 0.9**	**−0.2 ± 1.8**
Grazer		Lichtenstein’s hartebeest		*Alcelaphus buselaphus lichtensteinii*	01	3.5	−0.6	−1.4
					02	4.0	0.8	2.3
					03	3.5	1.1	2.9
		Roan antelope		*Hippotragus equinus*	04	5.8	−0.6	2.2
					05	3.9	1.0	3.2
		Sable antelope		*Hippotragus niger kirkii*	06	4.3	−1.5	5.3
**Grazer mean values (n = 6)**						**3.9 ± 0.9**	**0.1 ± 1.0**	**2.6 ± 2.2**

### Carbon isotope values

The δ^13^C_enamel_ values spanned the full isotopic range of C_3_ and C_4_ vegetation as expected for herbivores in African ecosystems, from −16.9 to 1.1‰ (H(3) = 17.69, *p* < 0.001). Fauna exhibits median values of −14.8 ± 0.4‰ for primates, −13.9 ± 0.9‰ for omnivores, −14.5 ± 0.9‰ for browsers, 0.1 ± 1‰ for grazers, and −15.1‰ for the porcupine (Fig. 2b). Primates had similar values to omnivores (*p* = 0.164) and browsers (*p* = 0.886) but had significantly lower δ^13^C_enamel_ values than grazers (p = < 0.001). Within the primate group, chimpanzees ($\tilde{\mathrm{x}}$ = −14.9 ± 0.1‰) and baboons ($\tilde{\mathrm{x}}$ = −14.3 ± 0.4‰) showed similar median values in δ^13^C_enamel_, but baboons were more variable. The δ^13^C_enamel_ value of the red-tailed monkey fell within the range observed in chimpanzees whereas the red colobus monkey showed a slightly higher value, similar to those of baboons. Grazers were significantly higher in δ^13^C_enamel_ compared to any other group (*p* < 0.001); no significant difference was found between omnivores and browsers (*p* = 0.135).

### Oxygen isotope values

The δ^18^O_enamel_ values of all specimens ranged from −4.9 to 5.3‰ (H(3) = 19.45, *p* < 0.001) with median values of −1.0 ± 0.9‰ for primates, −2.9 ± 1.4‰ for omnivores, −0.2 ± 1.8‰ for browsers, 2.6 ± 1.6‰ for grazers, and 0.1‰ for the porcupine (Fig. 2c). Primates had significantly lower δ^18^O_enamel_ values compared to grazers (*p* = 0.006) and higher ones than omnivores (*p* = 0.025) but overlapped with browsers (*p* = 0.237). Within the primates, chimpanzees had the highest δ^18^O_enamel_ values ($\tilde{\mathrm{x}}$ = −0.1 ± 0.5‰) followed by baboons ($\tilde{\mathrm{x}}$ = −1.3 ± 0.8‰), the red-tailed monkey (1.3‰), and the red colobus monkey (1.9‰). Browsers had significantly higher δ^18^O_enamel_ values compared to omnivores (*p* = 0.001), and were higher compared to both browsers (*p* = 0.049) and omnivores (*p* < 0.001).

### Isotopic pairs and ecological niches

Isotopic niche overlap (%) and niche area (‰^2^) were calculated using paired isotope values to compare the different groups. A 40% standard ellipse overlap corrected for small sample size (SEA_C_) for δ^13^C_enamel_/δ^15^N_enamel_, δ^18^O_enamel_/δ^15^N_enamel_, and δ^13^C_enamel_/δ^18^O_enamel_ was used in this study (Fig. 3; for details, see section Statistical Analysis; for data, including 95% SEA_C_ calculations, see Table S2). For primates, SEA_C_ could only be calculated for baboons due to small sample size (< 5) in the other taxa^43^. For chimpanzees, convex hulls are shown to visualize their ecological niche, and individual data points for red-tailed and red colobus monkeys are shown to highlight the ecological position for each primate species within the Issa food web.

δ^13^C_enamel_/δ^15^N_enamel_ isotope niche (Fig. 3a*)*: Baboons overlap with all other groups except the grazers and showed the largest overlap with omnivores (0.3‰^2^), accounting for 51.7% of the baboons’ SEA_C_, and 5.5% of the omnivores’ SEA_C_. This is followed by a < 0.1‰^2^ overlap with browsers, representing 1.3% of the primates’ SEA_C_ and 0.3% of the browsers’ SEA_C_. Omnivores, in turn, overlapped with browsers by 1.6‰^2^, corresponding to 26.9 and 64.3% of their respective SEA_C_. Grazers, however, showed no overlap with any other group and occupied a distinct δ^13^C_enamel_/δ^15^N_enamel_ isotopic niche.

δ^18^O_enamel_/δ^15^N_enamel_ isotope niche (Fig. 3b*)*: Baboons overlapped with no other groups. Omnivores, in turn, overlapped with browsers by 0.4‰^2^, corresponding to 6.7% and 4.1% of their respective SEA_C_. Grazers did not overlap with omnivores and baboons but showed an overlap of 0.2‰^2^ with browsers in δ^18^O_enamel_/δ^15^N_enamel_ isotopic niche, accounting for 3.0 and 3.6% of their respective niche space.

δ^13^C_enamel_/δ^18^O_enamel_ isotope niche (Fig. 3c*)*: Baboons overlapped with all other groups except the grazers and overlap the most with browsers (1.1‰^2^), accounting for 69.4% of the primates’ SEA_C_ and 15.5% of the browsers’ SEA_C_. This is followed by a 0.3‰^2^ overlap with omnivores, representing 45.3% of the baboons’ SEA_C_ and 14.0% of the omnivores’ SEA_C_. Omnivores, in turn, overlap with browsers by 0.4‰^2^. corresponding to 9.1% and 6.6% of their respective SEA_C_. Grazers, however, show no overlap with any other group and occupy a distinct δ^13^C_enamel_/δ^18^O_enamel_ isotopic niche.

## Discussion

Chimpanzees had the lowest median δ^15^N_enamel_ value (3.7‰) of all analyzed primate species, which is significantly lower than those of sympatric baboons and comparable only to local herbivores grazing in the miombo woodland and grasslands. These low δ^15^N_enamel_ values agree with low hair δ^15^N data from Issa chimpanzees, which averaged 4.1 ± 0.4‰^35^, and thus fall within the 1σ range of the enamel values. Issa baboons also feed on woodland trees and spend a greater proportion of their time feeding on plants in miombo biomes compared to chimpanzees^44–46^. Given that the overall vegetation at Issa has an average δ^15^N value of 2.8‰ (*n* = 31; Fig. S4a)^35^, even a conservative enrichment factor between diet and tooth enamel of + 2.3‰ (from Leichliter et al.^13^) would yield expected chimpanzee δ^15^N values of approximately 5‰. The substantially lower δ^15^N value observed in Issa chimpanzees therefore cannot be explained by the plant component of their diet alone, indicating the inclusion of an additional dietary source with substantially lower δ^15^N than the local plants.

Analyses of whole bodies of *M. subhyalinus* soldiers and workers from 12 mounds at Issa revealed a low δ^15^N value ($\tilde{\mathrm{x}}$ = 0.3 ± 0.9‰; *n* = 47)^37,47^, which is on average 2.0‰ lower than plants commonly eaten by chimpanzees (Fig. 3a). By combining quantitative observational data with nitrogen content (%N) from these studies, we can estimate the contribution of termites to the chimpanzee protein budget. Termites’ high crude protein relative to plant-based resources indicated that their inclusion in the diet can account for a substantial proportion of dietary nitrogen, possibly explaining the low δ^15^N_enamel_ value observed in Issa chimpanzees (Table 2). Observational data document that chimpanzees spend only around 4% of their feeding time fishing termites^48^. However, despite the proportionally small amount of time devoted to termite fishing, the high N content and digestibility of the termites indicate that they make up ∼41% of the Issa chimpanzee’s protein intake (Table 2).

Our isotopic data can provide additional quantitative constrains on the relative importance of the different nitrogen sources for chimpanzees. For example, using a simple linear mixing model: δ^15^N_enamel_ = f_termites_ * δ^15^N_termites_ + (1 - f_termites_) * δ^15^N_plants_ + ε, where δ^15^N_termites_ = 0.3‰, δ^15^N_plants_ = 2.3‰, and the trophic enrichment factor ε = 2.3‰, we can estimate the contribution of termites to dietary nitrogen. To reach the observed δ^15^N_enamel_ value of 3.7‰ in chimpanzees, termites would need to contribute approximately 50% of the chimpanzees’ nitrogen intake. For larger enrichment factors, as have been documented for enamel in experimental animals^13^, the contribution of termites to dietary nitrogen would need to be even higher. For example, considering a +3.3‰ enrichment, termites would need to account for nearly 95% of dietary nitrogen. If vertebrate meat is also consumed, which would result in enrichment in ^15^N, the contribution of termites to the overall nitrogen budget would need to be extremely high to account for the low δ^15^N_enamel_ values observed in Issa chimpanzees.

These calculations demonstrate that the inclusion of δ¹⁵N-depleted, nitrogen-rich termites is essential to explain the low δ¹⁵N_enamel_ values observed in Issa chimpanzees. The high contribution of termites to their protein budget may reflect the greater bioavailability of protein relative to plants, and/or small uncertainties in the direct observational estimates of the proportion of termites in the diet. Based on our conservative enrichment factor of 2.3‰ and the median published δ^15^N values of termites at Issa (Fig. 3a)^47^, we estimate that an exclusive termite-feeder would have an δ^15^N_enamel_ value of ≈ 2.6 ± 0.9‰. For comparison, we included δ^15^N_enamel_ data from two aardwolf specimens (*Proteles cristatus*), an obligate termite-feeder, which are with values of 2.5 and 3.2‰ consistent with this estimated isotope value (Fig. S1, Table S5). As the specimens originated from East London (South Africa) and thus a different ecosystem, this comparison should be interpreted with caution.

Interestingly, when comparing the combined isotopic niche (δ^13^C_enamel_/δ^15^N_enamel_, δ^15^N_enamel_/δ^18^O_enamel_, δ^13^C_enamel_/δ^18^O_enamel_), chimpanzees are clearly separated from the other primates and only overlap with browsing herbivores in the δ^13^C_enamel_/δ^18^O_enamel_ biplot.

Chimpanzees are close to the crested porcupine in the three isotopic dimensions. However, porcupines are obligative radicivores that dig for roots and underground storage organs, a behavior usually not observed in chimpanzees. It is important to note that a study published in 2007^49^, more than a decade prior to the habituation of the Issa community studied here, reported indirect signs of chimpanzees in the same area digging for underground storage organs. However, since habituation, this behavior has neither been directly observed nor has it appeared in tens of thousands of hours of footage from over 70 camera traps across the study area. The previously reported digging holes were therefore most likely attributable to other animals, to human activity in the area, or potentially a unique behavior of a neighboring chimpanzee community. This observation strongly supports our argument for a substantial contribution of termites to the N intake of chimpanzees.

Isotope data thus provide meaningful insights into the nutritional importance of food items that constitute a small fraction of the foraging budget, such as protein-rich insects, but whose intake is difficult to quantify through direct observation. Given that termite exploitation in chimpanzees across Africa is associated with tool-use techniques^50^, δ^15^N_enamel_ analysis may also shed light on early hominin tool use. As for chimpanzees, termites may have supplied early hominins with a protein rich resource comparable to meat, but one that is more easily acquired with lower risk^51^.

**Table 2:** Observed feeding time, nitrogen content, dietary protein contribution, and δ¹⁵N values of foods consumed by baboons and chimpanzees at Issa. Feeding time (expressed as a percentage of total observed feeding activity) was modified after Schulze et al.^48^ for general food items and termite fishing activities after Giuliano^48^. Nitrogen content (%) was determined using data from locally collected plants^35^ and termites^47^ and from globally sourced mushrooms^52^. Dietary protein is calculated using observed feeding time weighted by nitrogen content for each resource type. δ¹⁵N values (‰) are reported where measurements were available. Termites (*Macrotermes subhyalinus*) provide a substantial portion of dietary nitrogen for chimpanzees despite representing only a small fraction of observed feeding time.

	Observed feeding time (%)	*N*-content (%)	Dietary protein (%)	δ^15^*N* (‰ vs. AIR)
**Baboon**				
Fruits	19	0.8	7	4.2
Flowers	3	–	1	–
Leaves	5	1.6	4	1.3
Invertebrates	10	–	47	–
Seeds/Pods	26	–	9	–
Grasses	5	1.6	4	3.4
Roots	18	–	6	–
Mushrooms	11	–	22	–
**Chimpanzee**				
Fruits	53	0.8	32	4.2
Flowers	11	–	7	–
Leaves	12	1.6	14	1.3
Seeds/Pods	14	–	8	–
Termites	4	10.5	32	0.3
Other Invertebrates	2	–	–	–
Mushrooms	2	4.5	7	–

While termites are the primary invertebrates consumed by Issa chimpanzees, baboons consume a wider variety of invertebrates, which together account for approximately 10% of their observed feeding time^36^ (Table 2). Although isotope data for these invertebrates are not yet available from Issa, data from Serengeti National Park, ca. 700 km NE of Issa in northern Tanzania, showed δ^15^N values ranging from 3.2 to 12.9‰^53^, thus generally much higher than those of *Macrotermes*. These invertebrate values typically fall within or even above the δ^15^N range of the overall vegetation at Issa. This pattern is also reflected in the high δ¹⁵N_enamel_ values of the mongoose (*Bdeogale crassicauda*), red-tailed monkey (*Cercopithecus ascanius*), and civet (*Nandinia binotata*), all of which consume a high proportion of insects and other invertebrates, while termites appear to form only a minor component of their diet^40^^,42,^^48,^^54^.

Additionally, baboons consumed different foods, including large amounts of grasses and roots, which are rare or absent in the chimpanzees’ diet. The δ^15^N value of grass (3.4‰) at Issa falls close to the mean value of the general vegetation (3.0‰). δ^15^N data for roots, seedpods, and mushrooms are not yet available. Consumption of vertebrates and invertebrates (excluding termites) generally results in elevated δ^15^N_enamel_ values, thus the relatively low δ^15^N_enamel_ value observed in *Papio* remain unexpected^36^^,55^. Seeds and seedpods also contribute substantially (26%) to the diet, with baboons primarily feeding on miombo woodland (*Brachystegia* and *Julbernardia)* species, showing low δ^15^N values in leaves and fruits compared to the overall vegetation^35^^,36^. Roots, another important dietary component for baboons, contribute about 18% to the diet at Issa, based on observed feeding time^36^. Although δ^15^N values of these plant components at Issa are not yet available, data from Kruger National Park (South Africa) show that roots generally have lower δ^15^N values compared to other plant organs across both C_3_ and C_4_ plants^22^. Supporting this, the δ^15^N_enamel_ value of the crested porcupine which feeds primarily on these roots and underground storage organs^55^ are low. Together, these observations suggest that the consumption of subterranean plant tissues can contribute to the low δ^15^N_enamel_ values observed in baboons. Future analyses of stable isotopes in roots and other plant components, in combination with behavioral data, could more directly identify the specific dietary resources responsible for these depleted δ¹⁵N signatures. However, while both chimpanzees and baboons are feeding on substantial amounts of low δ^15^N items they are separated by about 0.9‰ in our dataset. δ^15^N analysis of mushrooms at Issa may help explain observed differences in δ^15^N values between baboons and chimpanzees as they are consumed by both primate taxa but in different quantities^52,^^56^. Mushrooms, the fruiting bodies of fungi, are high in crude protein and are increasingly recommended as an alternative protein source to meat in human populations globally^57^. Mushrooms have highly variable isotopic compositions, with δ^15^N values ranging globally from − 7.1 to 21.8‰ depending on species^52^. Mushrooms, are a common food source for primates at Issa and contribute up ca. 10% to the baboons diet year-round, with a peak intake of up to 30% during the wet season^36^. In contrast, mushrooms make up only ca. 2% of diet of chimpanzees and red-tailed monkeys. Moreover, baboons consume a greater variety of fungal species compared to chimpanzees^36^, including a variety of large edible ectomycorrhizal fungi, such as different species of *Cantharellus*, *Lactifluus* and *Russula*^36^. Thus, mushroom consumption may substantially contribute to the mineral-bound nitrogen in the enamel of baboons. Isotopic analyses of a variety of fungal fruiting bodies collected at Issa Valley are currently underway and will help clarify whether the consumption of these resources contributes to the higher δ^15^N_enamel_ values observed in baboons compared to chimpanzees at this site, or if the observed values are just a result of substantially lower termite consumption. Mushrooms may have played an important role in primate diets, both as a nutritional resource and as a source of (self-) medication^52,^^58^. Integrating mycophagy into the dietary ecology of extant primates will help illuminate the role of this third, and often understudied, food source.

Given our small sample size for red-tailed (*n* = 1) and red colobus (*n* = 1) monkeys, we cannot make broad generalizations about their dietary behavior. Nevertheless, we can propose factors that may account for the observed variation in isotope ratios. Both individuals exhibited higher δ^15^N_enamel_ values than the larger primates in this study (Fig. 2a). While both chimpanzees and red-tailed monkeys feed on forest fruits and leaves in similar amounts^59^, red-tailed monkeys at Issa have been observed to hunt and consume birds^60^, as well as a variable amount of invertebrates, which could contribute to the higher observed δ^15^N_enamel_ values in this taxon. Red colobus monkeys occupy the high canopy and are mainly folivorous, supplementing their diet with seeds, fruit, bark, and invertebrates^61^. While the smaller primates at Issa differ from chimpanzees and baboons in δ^15^N_enamel_, we do not see similar differences in δ^13^C_enamel_ or δ^18^O_enamel_, possibly pointing to a similar use of plant and water resources.

Regarding intra-fauna comparison from the Issa Valley, we observed isotope niche differentiation between browsers, grazers, and omnivores. Primates exhibited no overlap with grazers in any of the isotope biplots (Fig. 3), suggesting that they primarily foraged in the C_3_ dominated woodland habitats, thereby avoiding direct competition with grazers.

## Conclusion

This study focused on the mosaic savanna-woodland landscape in the Issa valley, an environment broadly similar to those in which early hominins may have evolved. We used chimpanzees and baboons as modern analogues to investigate how isotope data (δ^13^C_enamel_, δ^15^N_enamel_, δ^18^O_enamel_) reflect the ecological position of these primates. We integrated field observations and isotopic data, providing important behavioral context not available for fossil hominids. We found that Issa’s primates prefer C_3_ resources and occupy an isotopic niche that is distinct from that of grazers, overlapping primarily with browsers and, to a lesser extent, with omnivores.

Our isotope data further reveal a distinct isotopic niche for chimpanzees in both δ¹³C_enamel_/δ¹⁵N_enamel_ and δ¹⁵N_enamel_/δ¹⁸O_enamel_ space, indicating a dietary composition that differs markedly from that of the other primates or any other faunal groups. Chimpanzees consume dietary resources relatively low in δ^15^N, and observational data are essential to identify the specific dietary components. In all three isotope systems, chimpanzees cluster closely to the porcupine – a specialized radicivore – but the feeding behavior of the two taxa differs, as wild chimpanzees at Issa (or elsewhere) are not known to routinely dig for and eat roots or underground storage organs. Instead, their low δ¹⁵N_enamel_ values likely reflect a substantial contribution of termites to their nitrogen intake. Our estimates suggest that termites provided at least 50% of the Issa chimpanzees’ nitrogen requirements, making termite consumption the primary factor shaping their unique isotopic signature.

In contrary to the chimpanzees, the consumption of roots, seeds and seedpods could play an important role in driving baboons’ relatively low δ¹⁵N_enamel_ values, all items that are regularly consumed by baboons. However, specific seeds or seedpod data for miombo species are not available at Issa and we were limited to fruit and leave data, which show overall low δ^15^N values for our calculations. Also roots have not yet been analyzed for δ^15^N at Issa, but evidence from the δ^15^N_enamel_ values of the analyzed porcupine, as well as δ^15^N data of roots from South Africa^22^ suggest them as low in δ^15^N. In addition, the frequent consumption of mushrooms^36^ may contribute to the relatively higher δ^15^N_enamel_ values in baboons compared to chimpanzees. All dietary components would benefit of further isotope investigation to enable a more coherent breakdown of the contribution of individual dietary items to the nitrogen budget.

Additional research could include serial sampling of the same tooth, to evaluate seasonal variability in diet, particularity with respect to the consumption of nutrient-rich food resources termites in chimpanzees and mushrooms in baboons. Our findings from bulk enamel sampling provide a robust foundation for such work. Additionally, non-traditional isotope systems (e.g., calcium, strontium, zinc) are increasingly used to complement traditional stable isotopes analysis in reconstructing dietary variability and resource use. A forthcoming complementary study using the same specimens and enamel powders will examine these non-traditional isotopes to further investigate inter- and intra-species dietary variation at Issa.

Finally, our study analyzed only late-forming molars, which reflect diet after weaning. A comparison of early- and late- mineralizing teeth, using both traditional and non-traditional isotopic approaches, could provide valuable insight into nursing, weaning, and early-life dietary transitions in primates and early hominins.

We conclude that from our isotope analyses that termites, seeds, seedpods and roots are dietary items, that can contribute to the low δ^15^N_enamel_ values observed in primates. For baboons, observational data suggest that seeds and seedpods of miombo legumes and root consumption is the one of the primary factors shaping their isotopic niche, and they appear higher compared to our chimpanzees because of the frequent consumption of mushrooms. In contrast, the distinct isotopic niche of chimpanzees – well separated from primates – probably reflect the consumption of termites, an energy-rich dietary component that makes a substantial contribution to the overall protein budget. The inclusion of invertebrates may have supplied essential protein comparable to those from mammalian meat, potentially meeting the elevated energy demands associated with encephalization and contributing to the evolutionary success of the hominin lineage.

## Materials and methods

### The Issa Valley

The Issa Valley – located ca. 90 km east of Lake Tanganyika in western Tanzania – offers an ideal setting for environmental and dietary reconstructions due to its remote location (the nearest town, Uvinza, is about 70 km to the NW) and modest anthropogenic influence. For isotopic studies, such conditions are advantageous, as artificial fertilizers result in an δ^15^N-enriched groundwater and soil, potentially confounding natural signals^62^.

Situated at 1550 m above sea level and part of the Greater Mahale Ecosystem, the area encompasses a mosaic vegetation, including miombo woodland (characterized by the genera *Brachystegia* and *Julbernardia*), seasonally inundated grasslands, as well as riverine and thicket forests^31^. The relative abundance of plants in the surrounding areas are described by Moore^63^ and plant isotope data suggest that the savanna grasses at Issa include C_4_ species, while the riparian vegetation is dominated by C_3_ plants^35^ (Fig. S4). The region experiences two distinct seasons: a wet season from November to April, and a dry season from May to October, with dry months defined as those with less than 100 mm rainfall. Environmental data collected between 2009 and 2015 showed that the Issa Valley receives annual rainfall ranging from 930 to 1650 mm, and daily temperatures of the region range from 11 to 38°C^54,55^.

Issa is home to eight different primate species^64^, some of which (i.e. eastern chimpanzees, yellow baboons, and red-tailed monkeys (*Cercopithecus ascanius*)) have been the focus of continuous research in the Issa Valley since 2008, with group and focal follows of habituated individuals of baboons and red-tailed monkeys since 2012^54^, and chimpanzees since 2018^48^. Other diurnal primate species at Issa include blue monkeys (*Cercopithecus mitis*), ashy red colobus monkeys (*Piliocolobus tephrosceles*), and vervet monkeys (*Cholorocebus pygerythrus*)^64^. This diversity in primate species in a modern ecosystem with modest human influence resembles environmental conditions that some early hominins might have experienced in eastern Africa during the Plio-Pleistocene^65^^,66^.

### Materials

Tooth enamel from skeletal remains, primary isolated skulls, of 45 individuals representing 18 mammalian species, was sampled for isotopic analysis in July 2022 (Table 1). These specimens were opportunistically collected from naturally deceased individuals encountered between 2012 and 2021.

Given the focus on primate dietary reconstruction, we consider primates separate from the group of omnivores and visualize the different primate species individually in our data. The dataset includes a total of 15 non-human primates: four chimpanzees, nine baboons, one ashy red colobus monkey, and a red-tailed monkey. Sympatric fauna included for comparative purposes comprised eight omnivores (i.e. suids, a civet and mongooses), one radicivore (porcupine), as well as 15 browsers and six grazing bovids (Table 1).

We sampled from each specimen a small chip of tooth enamel. All samples were mechanically cleaned of surface contaminants, rinsed in ultrapure acetone, and air dried in a covered test tube before shipment to the Max Planck Institute for Chemistry (MPIC, Mainz, Germany). Here, if necessary, remaining dentin was carefully removed with a handheld Dremel multitool using a diamond drill tip at low speed (max. 2500 rpm). The enamel flakes were then crushed with an agate mortar and pestle, each sample yielding 15 to 77 mg of enamel powder. A small aliquot (< 0.2 mg) of this homogenized powder was used for δ^13^C_enamel_ and δ^18^O_enamel_ analysis, and another (< 10 mg) for δ^15^N_enamel_ analysis. All analyses were performed at the MPIC. Additionally, a split of ca. 10 mg was sent to collaborators at Ecole normale superieure de Lyon (Lyon, France) for complimentary calcium, magnesium, stable strontium and zinc isotope analyses of the same specimens.

### Methods

### δ^15^N_enamel_ analysis using the oxidation denitrification method

Mineral-bound δ^15^N_enamel_ was measured using the oxidation-denitrification method at the Organic Isotope Geochemistry Laboratory, MPIC, Germany following the protocols of Leichliter^13^. The method was originally introduced for marine archive analysis^67^^,68^and is routinely applied to measure δ^15^N in modern and fossil materials including corals, foraminifera, diatoms, and teeth. The method was first applied for analyzing δ^15^N_enamel_ by Leichliter^13^ using rodent teeth from a controlled feeding experiment, and then subsequently used to validate diet and trophic behavior reconstructions of wild mammals in natural African ecosystems^17^^,18^. Further studies of the δ^15^N_enamel_ values of fossil teeth showed that enamel is highly resistant to alteration under experimentally altered conditions in a laboratory study^69^, reconstructs diet in Pleistocene southeast Asian fossil assemblages^15^ and Pliocene southern African *Australopithecus* sp.^14^.

The powdered enamel samples were prepared for analysis by first removing potential metal oxides through a reductive cleaning with a dithionite solution in centrifuge tubes. Samples were transferred into glass vials and exogenous, non-mineral-bound organic matter was then removed using a potassium persulfate oxidation reagent (POR)^70^. The apatite matrix was subsequently demineralized with hydrochloric acid to free the mineral-bound organic N, followed by a second oxidation step using POR to convert liberated N to nitrate. The resulting nitrate was then converted to nitrous oxide (N_2_O) by the denitrifying bacteria *Pseudomonas chlororaphis*, which are grown, cultured, and harvested at MPIC following the protocols of Sigman et al. and Weigand et al.^68,71^.

δ^15^N_enamel_ measurements of the bacterially produced N_2_O were carried out using a custom-built multi-valve system designed to aggregate and separate N_2_O from residual CO_2_ in the gas flow using a series of water and CO_2_ traps coupled with a Thermo Fisher Scientific MAT 253-Plus gas-chromatography mass spectrometry after Sigman et al. and Weigand et al.^68,71^. Measurements were performed at a target load of 5 nmol for optimal precision, typically using 4.4 to 8.5 mg of untreated enamel. During chemical pretreatment, 10 to 55% of initial material mass was lost due to reagent reactions and handling, resulting in 2.4 to 6.9 mg pre-treated enamel powder. To validate measurement precision, out of the 45 specimens of our dataset, we measured 24 enamel samples in duplicates resulting in 69 individuals across three analytical batches.

To monitor instrumental and methodological precision, we included four in-house (see Table S1 for details) and four international standards. The in-house standards include two powdered enamel samples of Proboscideans (the modern elephant “AG-Lox” and fossil mammoth “Mammy”, respectively), and two modern coral powders (“CF-1” and “PO2”), which were each measured in triplicate in each analytical batch, resulting in a precision of 0.2‰ across all batches. Additionally, triplicates of the international standards USGS-65 (glycine) and USGS-40 (L-glutamic acid) were included during the oxidation step, as well as IAEA-NO3 (potassium nitrate) and USGS-34 (nitrate) during the bacteria injection step (at least 3 replicas in different concentrations). Inter-batch precision (± 1σ) for δ^15^N for USGS-40 (*n* = 9) was < 0.2‰ and for USGS-65 (*n* = 9) 0.1‰, respectively. Furthermore, we introduce 15 blank vials per batch during the demineralization to record the fraction of the blank and its δ^15^N value (< 0.3 nmol/ml; range − 39 to 3‰). The average blank contribution was ca. 1%. Moreover, two bacteria blank vials are analyzed in each batch. For details see Leichliter et al.^13^.

### δ^13^C_enamel_ and δ^18^O_enamel_ analyses using the cold-trap method

For δ^13^C_enamel_ and δ^18^O_enamel_ analyses, a small aliquot (70–120 µg) of untreated enamel powder was analyzed in the inorganic gas isotope geochemistry lab at the MPIC using the “cold trap method”^72^. A Thermo Scientific Gasbench II continuous flow interface unit with a pneumatically activated liquid N_2_ trap coupled with a Thermo Scientific Delta-V mass spectrometer was used. Weighted sample powder was automatically flushed with 5.0 grade helium (He) for 90 minutes before being acidified with > 99% H_3_PO_4_ at 70 °C in 12 ml Labco exetainer vials for another 90 minutes. The He carrier gas flushes the CO_2_ sample gas into the liquid N_2_ trap where it is cryogenically focused for 6 to 7 minutes. The accumulated sample gas is transported into the spectrometer resulting in one single sample peak. Two international standards (IAEA-603 and NBS-120c) and three in-house standards (Carrara marble “VICS” and the fossil tooth enamel standard “Mammy” and “Ag-Lox”, the same material used for δ^15^N_enamel_ measurements) are used to calculate analytical precision^72^. For internal precisions refer to table S4.

### Statistical analysis

Statistical analysis was conducted in R (version 4.3.1. R Core Team. 2024). Non-parametric statistics were used, as normality could not be assumed due to the small sample size of many groups. Statistical significance between isotopic groups was calculated using a Kruskal-Wallis Test followed by post-hoc Dunn`s test with a significance level of *p* = 0.05, Spearman’s rank correlation coefficient (r_s_) is given for correlations. Outliers were identified using Tukey’s Fence (k = 1.5), which were then excluded for statistical significance test and typically median values ($\tilde{\mathrm{x}}$) are stated for each group. p-values and H-statistics are reported in the main text while z-scores are reported in Table S3.

Furthermore, multivariate isotope comparisons were performed using the Stable Isotopes Bayesian Ellipses (SIBER) package^43^ and the statistical code after Turner^73^. For all groups (baboons, omnivores, browsers, grazers) isotopic niche analysis was performed for δ^15^N_enamel_, δ^13^C_enamel_, δ^18^O_enamel_ with estimated standard ellipses areas corrected for sample size (SEA_C_). SEA_C_ values for 40% ellipses were calculated and the proportional niche overlap (%) is reported in the text. Additionally, calculations for 40% and 95% ellipses including proportional and total overlap (‰^2^) were calculated and are reported in table S2. Additionally centroid distance between groups was calculated using Layman Metrics^74^ and a Hotelling T2 test was performed.

## Supplementary Information

Below is the link to the electronic supplementary material.

Supplementary Material 1

Supplementary Material 2

## Data Availability

All data are available in the main text or the supplementary material.
