# Supplementary material for: Multi-isotope analysis reconstructs termite feeding in chimpanzees
Source: Sci Rep. 2026 Apr 28;16:14026. doi: 10.1038/s41598-026-45049-4 (PMC13134952; doi:10.1038/s41598-026-45049-4)
Supplement: Supplementary file 2 — Supplementary Material 2 [file 41598_2026_45049_MOESM2_ESM.pdf]

Supplementary information for

Multi-isotope analysis reconstructs termite feeding in chimpanzees

Sven Brömme[1*], Vicky M. Oelze[2], Alfredo Martínez-García[3], Jennifer N. Leichliter[1], Gerald H. Haug[3,4], Hubert B. Vonhof[3], Fiona A. Stewart[5,6], Alex K. Piel[5,6], Tina Lüdecke[1*]

[1]Emmy Noether Group for Hominin Meat Consumption, Max Planck Institute for Chemistry, Mainz, Germany

[2]Department of Anthropology, University of Santa Cruz, Santa Cruz, CA, USA

[3]Department of Climate Geochemistry, Max Planck Institute for Chemistry, Mainz, Germany

[4]Department of Earth Sciences, ETH Zürich, Zürich, Switzerland

[5]Department of Human Origins, Max Planck Institute for Evolutionary Anthropology, Leipzig

[6]Department of Anthropology, University College London, London, United Kingdom

*Corresponding authors: Sven Brömme (Sven.broemme@mpic.de) and Tina Lüdecke (Tina.luedecke@mpic.de)

Tooth formation and nursing effect

During sample preparation, tooth enamel fragments were chipped off from individual teeth and crushed into powder. The resulting powder may represent hydroxyapatite formed over varying periods within an individual's life as teeth mineralize gradually over the course of several months to years. For example, in both chimpanzees and baboons, first molars (M1s) begin mineralizing in utero or shortly after birth and therefore during breastmilk consumption.

The consumption of (breast) milk can be detected across all three here studied isotope systems ($\delta^{13}C$, $\delta^{15}N$, $\delta^{18}O$)[1,2]. Isotopic enrichment in $\delta^{15}N$ during breastfeeding, as infants consume milk synthesized from maternal tissues. This trophic level enrichment is evident in early-forming teeth that mineralize in utero or shortly after birth, during the nursing period. Consequently, $\delta^{15}N_{enamel}$ values in these teeth are elevated compared those forming post-weaning[1,2]. This nursing-effect can also result in higher $\delta^{13}C_{enamel}$ and $\delta^{18}O_{enamel}$ values[3,4]. However, limitations in detecting breastfeeding in oxygen isotopes have been reported, for instance in enamel from orangutans, which showed enriched $\delta^{18}O_{enamel}$ values well after individuals were completely weaned[5].

To avoid the confounding influence of the nursing effect, our study focusses on late-forming permanent teeth, typically third molars (M3s), which mineralize after weaning and therefore reflect the adolescent diet. In chimpanzees, M3s mineralize between two and five years of age[6,7]. This approach enables reconstruction of dietary behaviors of individuals within their food web and provides a valuable reference for paleoenvironmental interpretations.

Thus, dietary behavior based on tooth enamel records the early life stages. Behaviors or skills acquired later, such as possible hunting developed or refined in later adulthood, would not be recorded in the isotopic composition of teeth. From an evolutionary perspective, the juvenile phase is particularly important, as key developmental processes, especially those related to brain growth and cognitive maturation, occur during this time.

Dietary niche reconstruction of herbivores and omnivores at Issa

Grazing herbivores at Issa occupied a distinct isotopic niche in all three isotope systems, with no overlap with other groups. The only exception is a small (0.2%^{2}) overlap with browsers in $\delta^{15}N_{enamel}/\delta^{18}O_{enamel}$ space, corresponding to less than 4% of their respective isotope niches. This separation is primarily driven by the grazers' almost exclusive consumption of C_4 biomass with high $\delta^{13}C$ values, which also tend to exhibit lower $\delta^{15}N$ values in African ecosystems. Our data are consistent with published $\delta^{15}N$ values for C_4 grass from Issa ($\tilde{x}$ = 3.4‰, n = 1 , Fig. S4a)[8] and Kruger National Park ($\tilde{x}$ = 3.5 ± 2.4‰, n = 510)[9]. Based on isotope analyses, primates were well separated from the grazers in $\delta^{13}C_{enamel}$, which suggests that C_4 grasses are not an essential part of their diet, even though these resources are available at Issa.

Browsing herbivores shared 64.3% (1.6‰^{2}) of their $\delta^{13}C_{enamel}/\delta^{15}N_{enamel}$ space with omnivores, suggesting a broad overlap in C_3 resource use. In $\delta^{18}O_{enamel}/\delta^{15}N_{enamel}$ space, browsers occupied a niche that overlaps minimally with omnivores and grazers (both ca. 4%). Omnivores showed a small overlap (6%) with browsers but are otherwise separated from all other groups.

Omnivores at Issa showed an overlap with browsers in all niche spaces: 26% in $\delta^{13}C_{enamel}/\delta^{15}N_{enamel}$, 7% in $\delta^{18}O_{enamel}/\delta^{15}N_{enamel}$, and 9% in $\delta^{13}C_{enamel}/\delta^{18}O_{enamel}$ respectively. Mongooses feed on ca. 90% invertebrates[10] and our data confirms that they target different types of insects instead of termites, resulting in a higher $\delta^{15}N_{enamel}$ isotopic signature. This is further supported by the values of the red-tailed monkeys, that also feed on up to 70% insects with a $\delta^{15}N_{enamel}$ value close to those of the mongooses.

The porcupine plots close to the chimpanzees in all isotope spaces and is well separated from any other faunal group in $\delta^{13}C_{enamel}/\delta^{15}N_{enamel}$ and $\delta^{18}O_{enamel}/\delta^{15}N_{enamel}$ space, probably due to the fact that it is the only species that feeds primarily on subterranean plant tissues[10]. In $\delta^{13}C_{enamel}/\delta^{18}O_{enamel}$ space, the porcupine plots near the center of the browsers' SEA_C, which indicates that primarily C_3 plants were consumed.

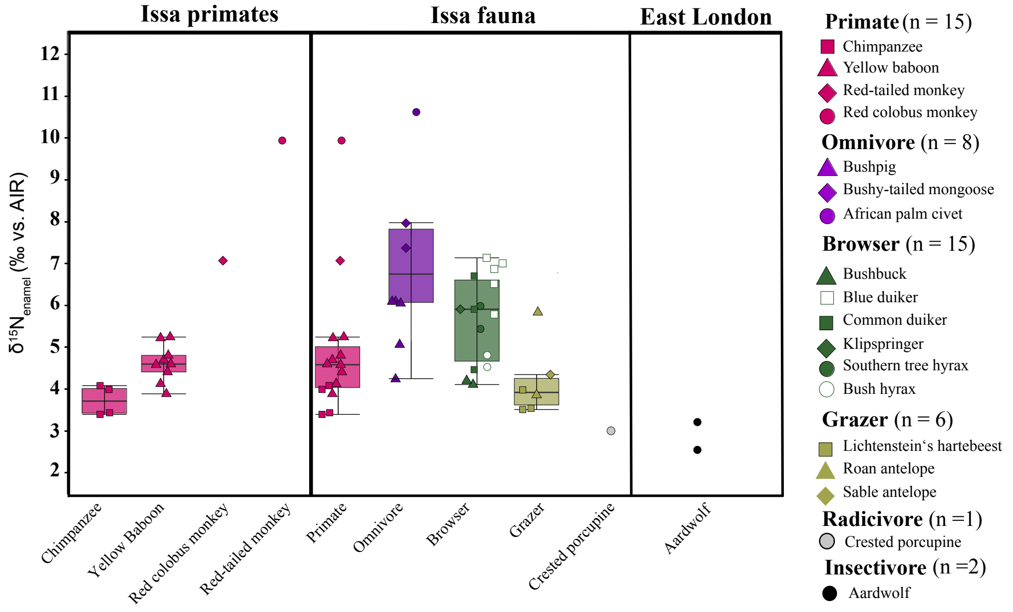

Figure S1: Isotopic values of the Issa Valley fauna and Aardwolves from East London (South Africa). Boxplots on the right show values grouped by ecological category, while those on the left display only the primate data, separated by taxon for better visualization. Boxplots show interquartile ranges with medians indicated as solid lines. The Aardwolf datapoint represent a $\delta^{15}N$ value for exclusive termite feeding despite the different locality.

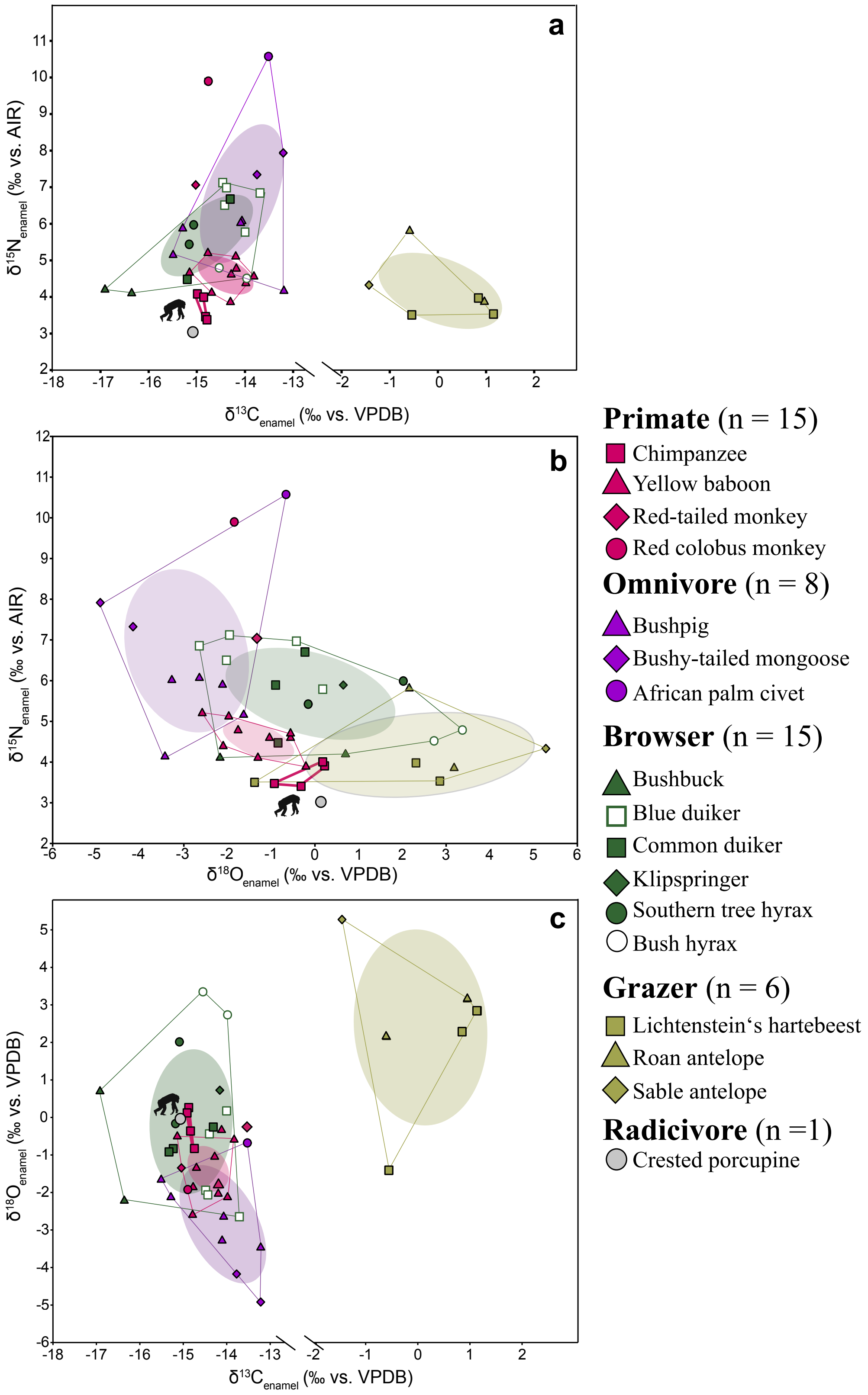

Figure S2: Biplots of (a) $\delta^{15}N_{enamel}$ vs. $\delta^{13}C_{enamel}$, (b) $\delta^{15}N_{enamel}$ vs. $\delta^{18}O_{enamel}$, and (c) $\delta^{18}O_{enamel}$ vs. $\delta^{13}C_{enamel}$ of Issa Valley fauna, following Fig. 3 in the main text, but including grazers. Ellipses indicate 40% SEA_C, convex hulls encompass full variation for each group (lines). Due to limited sample size (n < 5 of most primate taxa, SEA_C was calculated only for baboons, and the chimpanzees' isotopic niche is presented as convex hulls only. Individual data points of red-tailed and red colobus monkeys are displayed but excluded from the statistical analysis. Note the breaks in $\delta^{13}C_{enamel}$ axes.

Canopy effect

The combined interpretation of carbon and oxygen isotopes can also be used to investigate environmental effects such as the canopy effect. This is especially relevant for primates inhabiting multi-story forests, where leaves vary in light exposure, water availability, and toughness. Rather than being caused by elevation per se, the canopy effect arises from vertical gradients in light and humidity within the forest structure, which influence $\delta^{13}C$, $\delta^{18}O$, and $\delta^{15}N$ values of plant organs.

Specifically, leaves exposed to higher light intensity in the upper canopy experience greater water stress, resulting in elevated $\delta^{18}O$ values. In contrast, increased canopy cover and shading lower in the forest reduce photosynthetic rates, producing depleted $\delta^{13}C$ and $\delta^{18}O$ values due to limited sunlight and increased recycling of carbon dioxide within the understory[11-13]. Thus, the isotopic composition of primate enamel can reflect not only dietary preferences but also the vertical foraging niche within forest environments.

Nitrogen content

The median nitrogen content in tooth enamel was $\bar{x} = 6.6 \pm 2.0$ nmol/mg, and ranged from 2.8 to 10.7 nmol/mg (Fig. S3). These values agree well with published N contents of both modern and fossil enamel[14-17]. No correlation was observed between N content and $\delta^{15}N_{enamel}$ across the entire Issa sample ($r_s = 0.06$, $p = 0.654$), nor within any of the individual groups (Fig. S1).

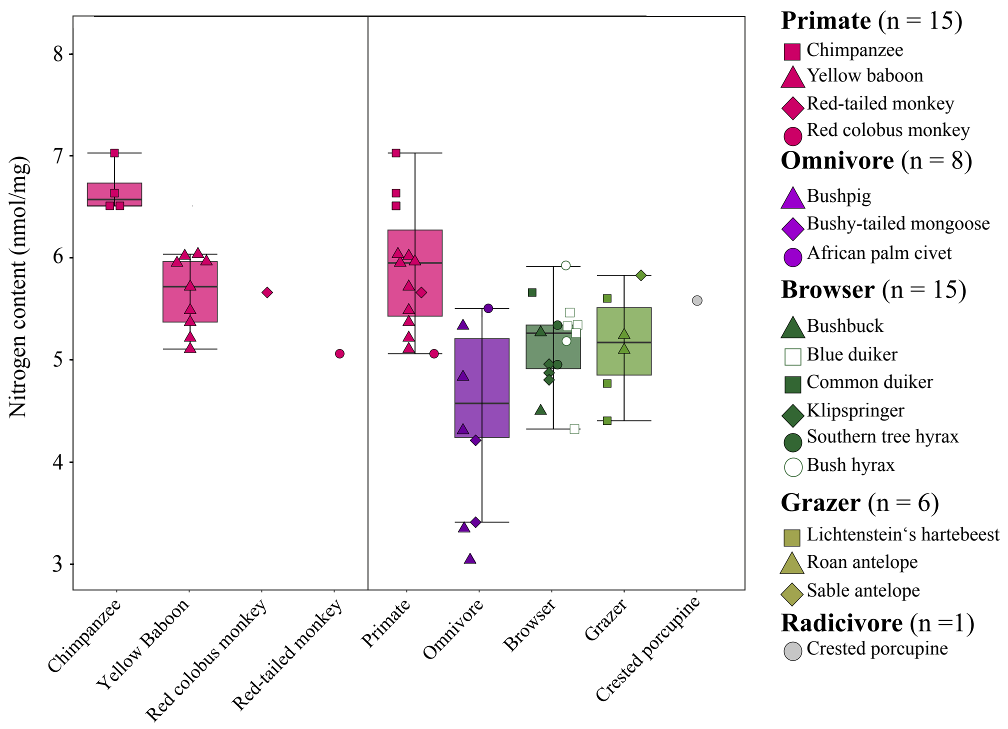

Figure S3: Nitrogen content of the Issa Valles fauna. Boxplots on the right show values grouped by ecological category, while those on the left display the same primate data, shown separated by taxon for better resolution. Chimpanzee enamel has a higher N content compared to the other groups. Primates show generally a higher N content compared to other ecological groups. N contents of enamel of Issa fauna agrees well with the N contents of modern and fossil enamel across Africa[14-17].

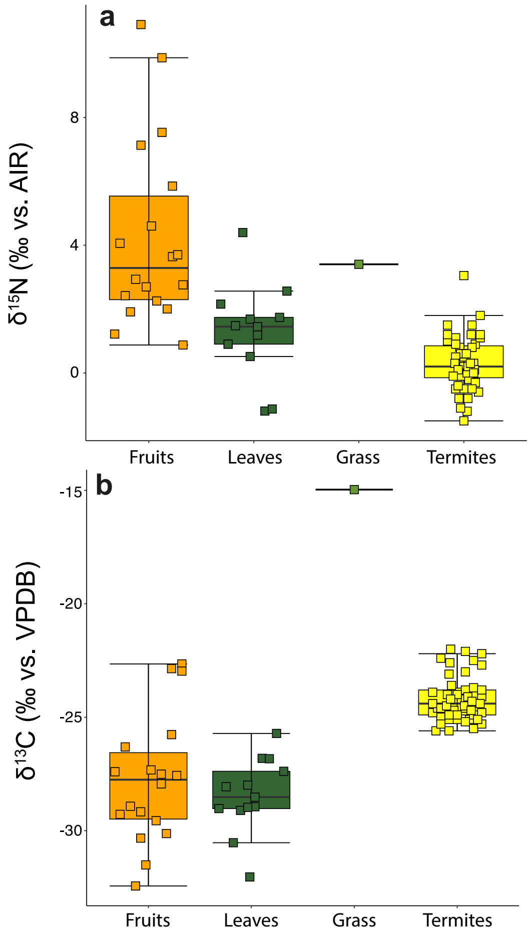

Figure S4: Published nitrogen (a) and stable carbon (b) isotope values of Issa Valley plant organs (from van Casteren et al.[8]) and termites (from Philips et al.[18]). Fruits show a greater variability in $\delta^{15}N$ and $\delta^{13}C$ values compared to leaves. C_4 grass is clearly separated from C_3 plants in $\delta^{13}C$, but not $\delta^{15}N$. Termites show lower $\delta^{15}N$ values than plant material and $\delta^{13}C$ values are generally higher compared to fruit and leaves, but lower compared to grasses.

References

1 Swindler, D. R. & Meekins, D. Dental development of the permanent mandibular teeth in the baboon, Papio cynocephalus. *Am. J. Hum. Biol.* **3**, 571-580, doi:https://doi.org/10.1002/ajhb.1310030606 (1991).

2 Smith, T. M. *et al.* Permanent signatures of birth and nursing initiation are chemically recorded in teeth. *J. Archaeol. Sci.* **140**, 105564, doi:https://doi.org/10.1016/j.jas.2022.105564 (2022).

3 Chinique de Armas, Y., Mavridou, A.-M., Garcell Domínguez, J., Hanson, K. & Laffoon, J. Tracking breastfeeding and weaning practices in ancient populations by combining carbon, nitrogen and oxygen stable isotopes from multiple non-adult tissues. *PLOS ONE* **17**, e0262435, doi:https://doi.org/10.1371/journal.pone.0262435 (2022).

4 Fuller, B. T., Fuller, J. L., Harris, D. A. & Hedges, R. E. M. Detection of breastfeeding and weaning in modern human infants with carbon and nitrogen stable isotope ratios. *Am. J. Phys. Anthropol.* **129**, 279-293, doi:https://doi.org/10.1002/ajpa.20249 (2006).

5 Smith, T. M. *et al.* Oxygen isotopes in orangutan teeth reveal recent and ancient climate variation. *eLife* **12**, RP90217, doi:https://doi.org/10.7554/eLife.90217.3 (2024).

6 Reid, D. J., Schwartz, G. T., Dean, C. & Chandrasekera, M. S. A histological reconstruction of dental development in the common chimpanzee, Pan troglodytes. *J Hum Evol* **35**, 427-448, doi:https://doi.org/10.1006/jhev.1998.0248 (1998).
7 Kuykendall, K. L. Dental development in chimpanzees (Pan troglodytes): The timing of tooth calcification stages. *Am. J. Phys. Anthropol.* **99**, 135-157, doi:https://doi.org/10.1002/(sici)1096-8644(199601)99:1%3C135::aid-ajpa8%3E3.0.co;2-# (1996).
8 van Casteren, A. *et al.* Food mechanical properties and isotopic signatures in forest versus savannah dwelling eastern chimpanzees. *Commun. Biol.* **1**, 109, doi:https://doi.org/10.1038/s42003%2D018%2D0115%2D6 (2018).
9 Codron, J. *et al.* Taxonomic, anatomical, and spatio-temporal variations in the stable carbon and nitrogen isotopic compositions of plants from an African savanna. *J. Archaeol. Sci.* **32**, 1757-1772, doi:https://doi.org/10.1016/j.jas.2005.06.006 (2005).
10 D'Ammando, G. *et al.* Ecological Drivers of Habitat Use by Meso Mammals in a Miombo Ecosystem in the Issa Valley, Tanzania. *Front. Ecol. Evol.* **Volume 10 - 2022**, doi:https://doi.org/10.3389/fevo.2022.773568 (2022).
11 da Silveira, L., Sternberg, L., Mulkey, S. S. & Joseph Wright, S. Oxygen isotope ratio stratification in a tropical moist forest. *Oecologia* **81**, 51-56, doi:https://doi.org/10.1007/bf00377009 (1989).
12 Lowry, B. E., Wittig, R. M., Pittermann, J. & Oelze, V. M. Stratigraphy of stable isotope ratios and leaf structure within an African rainforest canopy with implications for primate isotope ecology. *Scientific Reports* **11**, 14222, doi:https://doi.org/10.1038/s41598-021-93589-8 (2021).
13 Medina, E. & Minchin, P. Stratification of δ13C values of leaves in Amazonian rain forests. *Oecologia* **45**, 377-378, doi:https://doi.org/10.1007/BF00540209 (1980).
14 Leichliter, J. N. *et al.* Tooth enamel nitrogen isotope composition records trophic position: a tool for reconstructing food webs. *Commun. Biol.* **6**, 373, doi:https://doi.org/10.1038/s42003-023-04744-y (2023).
15 Leichliter, J. N. *et al.* Nitrogen isotopes in tooth enamel record diet and trophic level enrichment: results from a controlled feeding experiment. *Chem. Geol.* **563**, 120047, doi:https://doi.org/10.1016/j.chemgeo.2020.120047 (2021).
16 Lüdecke, T. *et al.* Carbon, nitrogen, and oxygen stable isotopes in modern tooth enamel: A case study from Gorongosa National Park, central Mozambique. *Front. Ecol. Evol.* **10**, doi:http://dx.doi.org/10.3389/fevo.2022.958032 (2022).
17 Lüdecke, T. *et al.* Australopithecus at Sterkfontein did not consume substantial mammalian meat. *Science* **387**, 309-314, doi:https://doi.org/10.1126/science.adq7315 (2025).
18 Phillips, S. *et al.* Limited evidence of C4 plant consumption in mound building Macrotermes termites from savanna woodland chimpanzee sites. *PLOS ONE* **16**, e0244685, doi:https://doi.org/10.1371/journal.pone.0244685 (2021).
